# A Combination of Magnetic Resonance Imaging Techniques to Localize the Dural Defect in a Case of Superficial Siderosis—A Case Report

**DOI:** 10.3390/medicines7060036

**Published:** 2020-06-25

**Authors:** Hiroyuki Katoh, Shuhei Shibukawa, Keiko Yamaguchi, Akihiko Hiyama, Tomohiko Horie, Masato Sato, Masahiko Watanabe

**Affiliations:** 1 Department of Orthopaedic Surgery, Surgical Science, Tokai University School of Medicine, Kanagawa 259-1193, Japan; keeeeeeko0430@yahoo.co.jp (K.Y.); a.hiyama@tokai-u.jp (A.H.); sato-m@is.icc.u-tokai.ac.jp (M.S.); masahiko@is.icc.u-tokai.ac.jp (M.W.); 2 Department of Radiology, Tokai University Hospital, Kanagawa 259-1193, Japan; shibu@tokai-u.jp (S.S.); tomohiko.horie@gmail.com (T.H.)

**Keywords:** superficial siderosis, spinal extradural arachnoid cyst, duropathy, magnetic resonance imaging

## Abstract

**Background:** Superficial siderosis is a progressively disabling disease caused by recurrent subarachnoid hemorrhage with accumulation of hemosiderin in the surface of the central nervous system. Although a wide variety of conditions may cause superficial siderosis, approximately half of the cases are reported to be associated with a defect in the ventral spinal dura mater, in which case treatment entails surgical repair of the defect. Here, we report a case of superficial siderosis and report on our method to pinpoint the dural defect using a combination of magnetic resonance imaging (MRI) techniques. **Methods and Results:** A 74-year-old female presented suffering from hearing loss and progressive ataxia over a duration of seven years. A T2-weighted MRI study revealed hypointensity in the superficial areas of the central nervous system, leading to the diagnosis of superficial siderosis, and the presence of a fluid-filled collection in the anterior spinal canal of C7 to T10 suggested that a dural defect was the cause of the repeated hemorrhage. A balanced turbo field echo (BTFE) MRI sequence revealed possible dural defects at T1–T2 and T5–T6, and a dynamic improved motion-sensitized driven-equilibrium steady-state free precession (dynamic iMSDE SSFP) sequence revealed an irregular flow of cerebrospinal fluid through the dura at the T5–T6 level. The dural defect was confirmed and sutured through a minimal T5–T6 laminectomy without neurological consequences, and the patient reported mild improvement in gait one year after surgery. **Conclusions:** A combination of MRI sequences provided the necessary information to confidently perform minimal surgery to repair the dural defect. We recommend coupling a balanced steady-state free precession (SSFP) sequence to provide high resolution, high contrast images of anatomical structures and a dynamic iMSDE SSFP sequence to confirm cerebrospinal fluid motion through the defect.

## 1. Introduction

Superficial siderosis of the central nervous system is an uncommon and often underdiagnosed disorder that is caused by repeated hemorrhages in the subarachnoid space. The bleeding episodes are small and individually asymptomatic, but the repetitive degradation of hemoglobin releases toxic heme into the cerebrospinal fluid (CSF), which chronically leads to deposition of hemosiderin in the subpial layers of the brain, cranial nerves, and spinal cord [1]. The superoxides produced by the free iron cause lipid peroxidation and membrane dysfunction, resulting in neuronal loss, reactive gliosis, and axonal demyelination [2]. The insidious progression of the condition often leaves patients suffering for years before being diagnosed with superficial siderosis, at which time patients often present with the clinical triad of hearing loss, cerebellar ataxia, and myelopathy [1].

Since any condition that causes repeated bleeding into the subarachnoid space could lead to superficial siderosis, the listed etiology in the literature is wide-ranging. An epidemiological survey of superficial siderosis in Japan found that duropathy was the leading cause in both men and women, accounting for approximately half of the patients [3]. Therefore, patients diagnosed with superficial siderosis need to be screened for the possibility of dural defects with magnetic resonance imaging (MRI).

The literature on spinal duropathy associated with superficial siderosis often describe a ventral dural defect, most often in the thoracic spine, with a longitudinally fluid-filled collection in the anterior aspect of the spinal canal. As to how duropathy in the spinal column could cause hemorrhage into the subarachnoid space, two theories have been proposed: the first cites bleeding from the bridging veins on the surface of the cerebellum that rupture due to brain sagging brought about by intracranial hypotension [4], and the second cites bleeding from the epidural veins around the dural defect [5].

By stopping the leakage of the CSF and thus the subarachnoid hemorrhage, surgical repair of the dural defect can stabilize the progression of neurological symptoms in most cases and may improve the symptoms associated with CSF hypovolemia [6]. Considering that the anterior fluid collection in the spinal column often spans multiple levels, the practical dilemma facing the surgeon is how to accurately determine the location of the dural defect. Here, we describe a combination of MRI techniques that allowed us to confidently determine the location of the dural defect and to perform a minimally invasive surgery to repair the lesion.

## 2. Case Report 

A 74-year-old female presented to our institute complaining of a floating sensation and difficulty in walking. The symptoms started seven years ago and progressively worsened, with accompanying right-sided hearing impairment from three years ago. Past medical history included mild diabetes, hyperlipidemia, and shingles. Her younger sister suffers from Sjogren syndrome, but her family history was otherwise unremarkable. 

Physical examinations revealed no cranial nerve function abnormalities and she did not suffer from dysarthria or dysphagia. Mild weakness of her legs was noted bilaterally, but deep tendon reflexes were normal. Fine touch, vibration, and proprioception sensory were all intact. Heel-shin testing revealed dymetria and tandem gait was noted to be unsteady, but she was negative for Romberg. A mild intention tremor was observed. 

**Figure 1:**
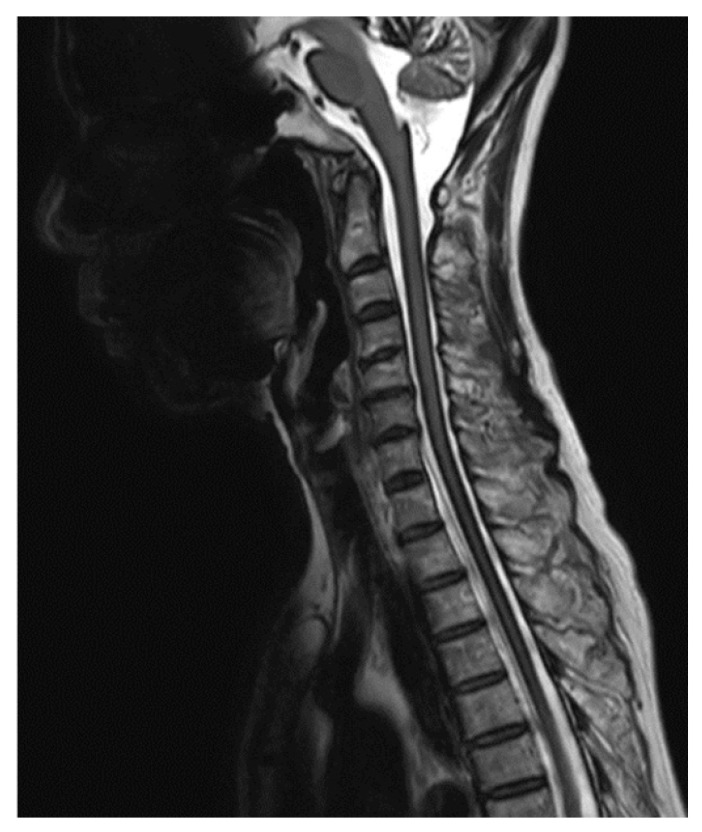
T2-weighted magnetic resonance imaging (MRI) of the cervical to upper thoracic spine reveals hypointensity in the superficial areas of the central nervous system which is characteristic of superficial siderosis. An anterior epidural fluid collection spanning the C7 to T10 levels is also visible.

T2-weighted MRI of the head revealed atrophy of the cerebellum and hypointensity in the superficial areas of the central nervous system, suggesting hemosiderin deposition and the possible diagnosis of superficial siderosis. There were no tumors, vascular abnormalities, amyloid angiopathy, or other lesions that could lead to subarachnoid hemorrhage. A standard whole-spine MRI confirmed superficial hypointensity of the entire spinal cord on T2-weighted images and revealed the presence of anterior epidural fluid collection spanning the C7 to T10 levels (Figure 1). A myelogram conducted to search for the dural defect revealed xanthochromia, but the extradural leak was not apparent under fluoroscopy. Furthermore, the contrast agent had already reached an equilibrium with the anterior epidural cyst by the time a CT was taken, and therefore, could not pinpoint the position of the dural defect. 

**Figure 2:**
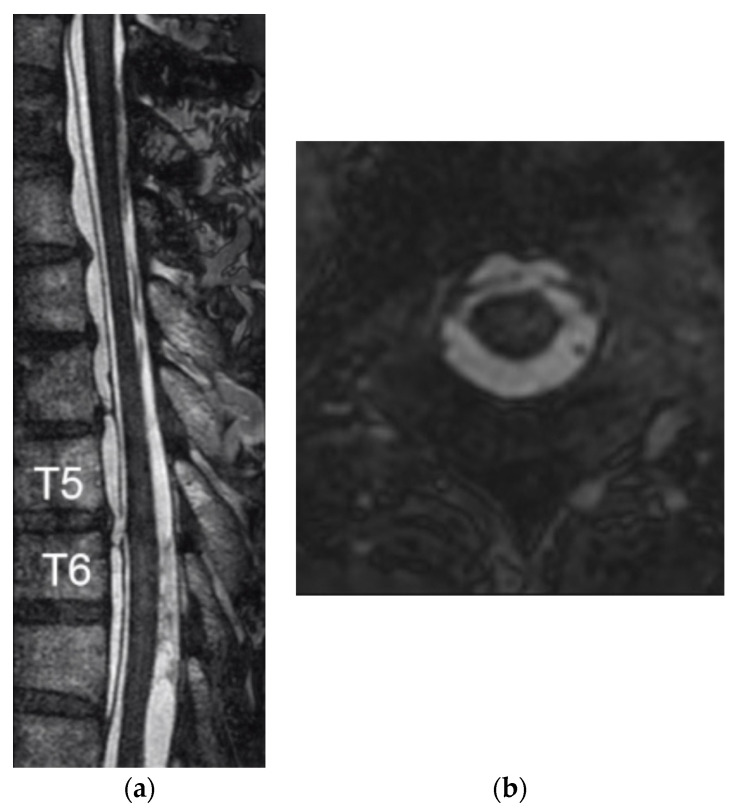
A balanced turbo field echo (BTFE) MRI sequence, which achieves rapid and high-resolution imaging of the spinal anatomical structures, reveals a gap in the anterior dura mater at the T5–T6 level. (**a**) sagittal-, and (**b**) axial-oriented images.

With the epidural lesion spanning 10 thoracic levels, blind exploratory surgery to search for the dural defect through multilevel laminectomies entails the risk of postoperative instability and kyphosis. In order to examine the integrity of the dura mater in more detail, the C7 to T10 level with the epidural fluid collection was studied using a 1.5-T clinical magnetic resonance scanner (Achieva R3.2, Philips Medical Systems, Best, The Netherlands). Rapid and high-resolution imaging of the spinal anatomical structures was performed utilizing a balanced turbo field echo (BTFE) sequence with the following parameters: repetition time (TR)/echo time (TE), 7.3/3.6 milliseconds (ms); field of view, 200 mm; matrix, 400; pixel size, 0.5 × 0.5 mm; slice thickness, 0.6 mm; and scan time, 8 min 23 s. Sagittal- and axial-oriented BTFE images revealed a possible gap in the anterior dura mater at the T5–T6 level (Figure 2 and Supplemental Video S1) along with the possibility of a small gap in the T1–T2 level. 

**Figure 3:**
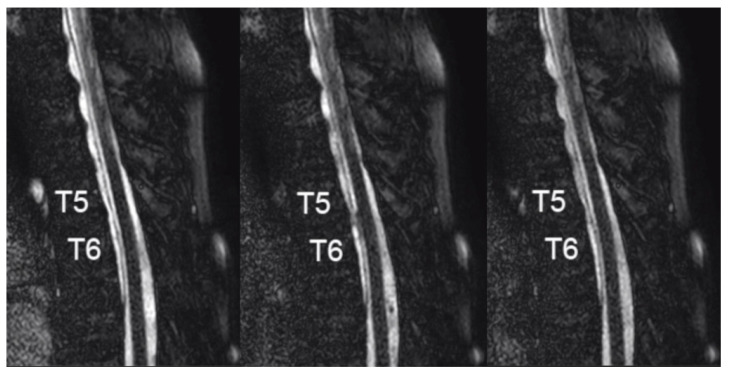
Images achieved by a dynamic improved motion-sensitized driven-equilibrium steady-state free precession (dynamic iMSDE SSFP) MRI sequence reveal an irregular flow of CSF through the dura at the T5–T6 level in the middle panel, confirming the position of the dural defect.

In order to confirm abnormal CSF motion through the dural defect, further analysis was conducted by using a dynamic improved motion-sensitized driven-equilibrium steady-state free precession (dynamic iMSDE SSFP) MRI sequence [7] with the following parameters: TR/TE, 4/2 ms; field of view, 220 mm; matrix, 208; pixel size, 1.06 × 1.09 mm; slice thickness, 8.0 mm; and scan time, 28 s. The acquired images revealed an irregular flow of CSF through the dura at the T5–T6 level, confirming the position of the dural defect (Figure 3 and Supplemental Video S2). 

**Figure 4:**
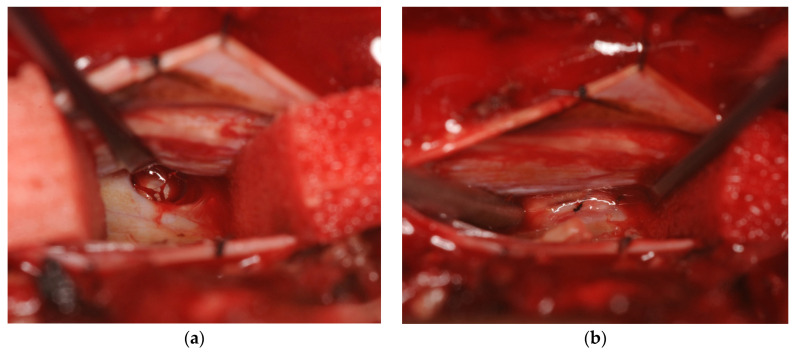
Intraoperative images of the T5–T6 dural defect before (**a**) and after (**b**) primary suture. In both images, caudal is on the left side of the image and the spinal cord is retracted superiorly.

Under motor evoked potential (MEP) monitoring, the patient underwent surgery to repair the dural defect. The dura was exposed through a T5, T6 laminectomy, and the dura mater as well as the thickened arachnoid was incised. The spinal cord was carefully retracted and a vertical dural defect measuring approximately 10 mm in cradiocaudal length was visualized in the midline ventral dura at the T5–T6 level (Figure 4a). The right T6 nerve root was cauterized and dissected to mobilize the spinal cord, and primary closure of the dural defect was performed with two stitches of 7-0 polypropylene (Ethicon, Somerville, NJ, USA) (Figure 4b).

**Figure 5:**
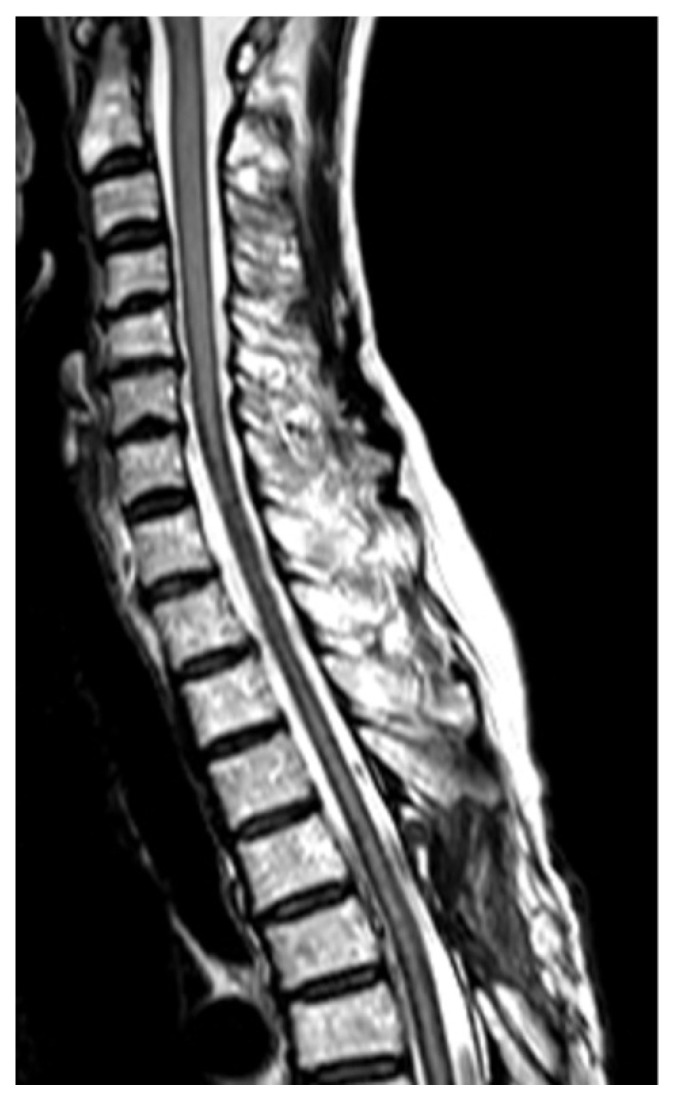
T2-weighted MRI of the cervical to upper thoracic spine taken three months after surgery confirms the resolution of the anterior epidural fluid collection.

The patient recovered from anesthesia without any worsening of motor deficits. An MRI taken three months after surgery confirmed the resolution of the anterior fluid collection but the hemosiderin deposition on the surface of the spinal cord was unchanged (Figure 5). One year after surgery, the patient reported mild improvement in gait, but she was still unable to walk more than 50 m without support, and many of her other symptoms were unchanged.

Written consent was given by the patient to present anonymized images and data in medical conferences and in a published case report.

## 3. Discussion

While some studies have attempted conservative treatment through steroids [8] or iron chelation [9], the majority of reported superficial siderosis cases with duropathy have been treated with surgical repair of the dural defect. Since Kumar et al. first described surgical repair of the defect [10], a major obstacle in the surgical planning for the condition has been the accurate localization of the dural defect. Kumar et al. described using dynamic CT myelography to identify the lesion [10], and the high spatial resolution and contrast brought about by the contrast medium made CT myelography the imaging method of choice for a long time. 

As a routine test in many facilities performing spine surgery, fluoroscopic myelography followed by CT imaging has been performed in many cases of superficial siderosis, but results were mixed. Similar to our case, the injected contrast medium often diffuses immediately up and down the epidural cavity through the small dural hole, making confident identification of the dural defect contingent on a well-timed dynamic CT scan. Some reports have suggested modified methods to increase the chances of achieving images that pinpoint the lesion location. Ryu et al. reported on a head-down, head-up protocol, which they claim showed a dural defect, but images were not provided [11]. Digital subtraction myelography has been shown to identify the lesion in multiple reports; Hoxworth et al. placed patients in the prone position [12], while Schieving et al. had patients in the lateral decubitus position [13], and both reported high success rates. Arishima et al. described a selective CT myelography procedure injecting small volumes of contrast medium through a lumbar spinal drainage tube with the patient in the prone position, but this was to confirm the dural hole identified by spinal endoscopy [14].

With the development of numerous MRI techniques for CSF-cisternography, MRI has now become the diagnostic tool of choice to visualize dural defects. However, the nomenclature for gradient echo sequences by MR manufacturers has made it difficult for clinicians to navigate the various techniques. Egawa et al. described using the Constructive Interference Steady State (CISS) sequence to identify the dural defect in the two cases [6], and Hiraka et al. located the dural defect utilizing a 3D Cube T2 sequence [15]. On the other hand, neither Takai et al., using a Fast Imaging Employing Steady-state Acquisition (FIESTA) MRI sequence [16], nor Arishima et al., using CISS and coherent oscillatory state acquisition for the manipulation of an image contrast (COSMIC) [14], could detect the dural defect with MRI. While the specific parameters vary among studies, balanced steady-state free precession (SSFP) sequences utilize a very short repetition time, employ radiofrequency pulses with a large flip angle, and balance gradients in all three directions [17]. With high spatial resolution along with high signal-to-noise and high contrast-to-noise ratios, these gradient echo sequences yield high signal of body fluids without using contrast material and can be used to visualize anatomical structures within the CSF space [18]. The BTFE sequence in this case depicted a gap in the ventral dura at the T5–T6 level, but another small defect was suspected in the T1–T2 level, prompting us to perform an additional MRI study. 

In past MRI studies of the spine, the use of kinematic MRI (cine-MRI) was reported to visualize the motion of CSF through a dural defect into a spinal extradural arachnoid cyst as a pulsating turbulent flow [19,20]. More recently, Ishibe et al. used a time-resolved three-directional phase-contrast MRI sequence termed time-spatial labeling inversion pulse magnetic resonance imaging (Time-SLIP MRI) to detect the communicating hole in cases of spinal extradural arachnoid cysts [21]. Time-SLIP MRI can detect abnormal flow in a designated area, but the process is complicated and time consuming. Its merit as well as demerit rise from the fact that it can selectively label and visualize the CSF motion of a targeted area, but it would be unsuitable to search for abnormal flow in a nonspecific and wide area, as was in this case with a suspected dural defect at T1–T2 as well as T5–T6. Therein lies the practicality of the novel sequence developed at our institution termed dynamic iMSDE SSFP, which visualizes the CSF motion in the entire acquired imaging plane in a relatively short period of time [7,22]. The dynamic iMSDE SSFP MRI demonstrated abnormal CSF flow through the dural defect at the T5–T6 level and excluded the suspected lesion at the T1–T2 level, allowing us to confidently perform minimal laminectomy and durotomy to repair the lesion. With recent further refinement of this method to three-dimensional imaging demonstrated by the visualization of CSF motion in the whole brain [23], its further utility in visualizing abnormal CSF flow in the spine is anticipated. 

While a number of studies have demonstrated the utility of MRI in identifying the dural defect in superficial siderosis and spinal extradural arachnoid cysts, some of the images provided by past reports have not been completely convincing. We feel that the combination of a BTFE sequence to provide high resolution anatomical information and a dynamic iMSDE SSFP to provide convincing imaging of abnormal CSF motion through the defect provides surgeons with the necessary information to confidently perform surgery to repair the dural defect.

## 4. Conclusions

In patients with superficial siderosis associated with epidural fluid collection anterior to the spinal cord, pinpointing the exact location of the dural defect is crucial to minimize surgical trauma and avoid instability or kyphosis brought about by multisegmental laminectomies. We recommend that this determination be made through a combination of MRI sequences: a balanced steady-state free precession (SSFP) sequence to provide high resolution, high contrast images of anatomical structures, and a sequence such as dynamic iMSDE SSFP to visualize CSF motion through the defect.

## Supplementary Materials

The following are available online at https://www.mdpi.com/2305-6320/7/6/36/s1, Video S1: Balanced turbo field echo (BTFE) MRI sequence images. Video S2: Dynamic improved motion-sensitized driven-equilibrium steady-state free precession (dynamic iMSDE SSFP) MRI sequence images.
